# Factors influencing vancomycin trough concentration in burn patients: a single center retrospective study

**DOI:** 10.3389/fphar.2024.1377930

**Published:** 2024-12-12

**Authors:** Yan Shi, Zongqi Yin, Qin Zhang, Lei Yi, Yi Dou

**Affiliations:** Department of Burn, Ruijin Hospital, Shanghai Jiaotong University School of Medicine, Shanghai, China

**Keywords:** vancomycin, trough concentration, burns, creatinine clearance, serum albumin

## Abstract

**Objective:**

To analyze factors influencing the vancomycin trough concentration in burn patients to provide a basis for the more rational use of vancomycin in these patients.

**Materials and methods:**

We collected the clinical data of adult burn patients treated with vancomycin in a Chinese hospital. Vancomycin was administered at a dosing regimen of 1.0 g q12 h. Patients were divided into a therapeutic group with vancomycin trough concentration in the target therapeutic range (10–20 μg/mL) and a subtherapeutic group with vancomycin trough concentration in the subtherapeutic range (<10 μg/mL).

**Results:**

The therapeutic group included 14 patients (17.5%), with an average trough concentration of 14.36 ± 2.82 μg/mL; the subtherapeutic group included 66 patients (82.5%), with an average trough concentration of 5.18 ± 2.77 μg/mL. The serum creatinine level was significantly higher in the therapeutic group (84.93 ± 47.26 μmol/L) than that in the subtherapeutic group (62.44 ± 14.49 μmol/L) (*p* < 0.01). Serum albumin levels were significantly lower in the therapeutic group (30.50 ± 2.28 g/L) than those in the subtherapeutic group (34.00 ± 6.22 g/L) (*p* < 0.05). Using receiver operating characteristic (ROC) curve analysis, for serum albumin, the area under the ROC curve (AUC) (95% confidence interval [CI]) was 0.67 (0.553, 0.788); the optimal cut-off point was 34.50 g/L (*p* = 0.046), the sensitivity was 0.379, and the specificity was 1.0. For creatinine clearance, the AUC (95% CI) was 0.72 (0.537, 0.902); the optimal cut-off point was 76.64 mL/min (*p* = 0.01), the sensitivity was 0.985, and the specificity was 0.5. The linear stepwise regression equation was as follows: trough concentration = 0.14 × age + 0.071 × serum creatinine −4.196.

**Conclusion:**

In this study, a high proportion of burn patients had a vancomycin trough concentration below the standard range. Serum creatinine clearance and albumin levels are important indicators for predicting whether the vancomycin trough concentration is within the standard range. Using a linear stepwise regression equation, the vancomycin trough concentration can be estimated using the patient’s age and serum creatinine level.

## 1 Introduction

Infection is an important cause of death in burn patients (Greenhalgh et al., 2007). The increased infection risk in patients with burn injury can be attributed to a variety of risk factors, including immune deficiency, loss of barrier function of the skin, bacterial translocation from the gastrointestinal tract, prolonged hospitalization, multiple invasive surgeries, prolonged intubation time, central venous catheter placement, and urinary catheter placement (Carter et al., 2015). *Staphylococcus aureus* is one of the leading bacterial causes of infection in burn patients (Dou et al., 2017). Vancomycin is a first-line drug for the treatment of *S. aureus*, especially methicillin-resistant *S. aureus* (Ayuthaya et al., 2020), and is widely used in clinical practice. However, the therapeutic window of vancomycin is narrow. A low vancomycin concentration can lead to treatment failure and the development of drug-resistant strains, and a high vancomycin concentration can increase the incidence of adverse reactions such as nephrotoxicity (Elyasi et al., 2012; Udy et al., 2011; Chu et al., 2016; Álvarez et al., 2017). Therefore, by assessing the pharmacokinetic/pharmacodynamic (PK/PD) parameters (steady-state blood trough concentration or AUC24) of vancomycin in patients (He et al., 2020), therapeutic drug monitoring of vancomycin can ensure that patients are being treated with the recommended and effective drug concentrations (Zhi-Kang et al., 2013; Nham et al., 2022; Chu et al., 2019). However, owing to unique pathophysiological changes in burn patients (Ortwine et al., 1900; Wu et al., 2017), the proportion of patients with a blood vancomycin concentration that reaches the target level is low (Schlobohm et al., 2021; Carter et al., 2015; Elligsen et al., 2011), and a high dose is often needed to achieve an effective drug concentration (Elder et al., 2017; Hill et al., 2018). There are numerous PK/PD studies on vancomycin for patients with other diseases (Aljutayli et al., 2020). However, owing to the differing pathological and physiological states in burn patients (Ortwine et al., 1900; Wu et al., 2017), including skin barrier loss, burn shock, tissue edema, high metabolic status, and high urine output, drug metabolism in these patients differs from that of other patients. Directly applying drug PK/PD data from patients with other diseases to burn patients may lead to treatment failure. Owing to the limited PK/PD research on drugs for burn patients, including vancomycin, it is unclear whether the concentrations of these drugs meet the standards when used in these patients. Therefore, in this study, we analyzed the factors influencing the blood vancomycin trough concentration in burn patients to provide a basis for the more rational use of vancomycin in the absence of timely blood concentration monitoring.

## 2 Materials and methods

### 2.1 Clinical data

This was a retrospective study. We included adult inpatients who received vancomycin and were admitted to the Department of Burns at Ruijin Hospital, Shanghai Jiaotong University School of Medicine from November 2018 to January 2021. We monitored the blood vancomycin concentration in each patient. The inclusion criteria were as follows: 1) age ≥18 years; 2) diagnosed with burn injury owing to fire or scalding water on admission to the hospital; 3) received an intravenous infusion of vancomycin 1.0 g q12 h and with blood vancomycin concentration detected after administration of the fifth vancomycin dose. The exclusion criteria were: 1) patients on dialysis and 2) pregnant or breastfeeding women. In a review of patients’ electronic medical records, we collected the following information for each patient: age, weight, height, burn area, area of third-degree burns, days between burn injury and drug administration, serum creatinine level before drug administration, serum albumin level before drug administration, Acute Physiology and Chronic Health Evaluation II (APACHE II) score, blood vancomycin trough concentration, tracheal incision, body mass index (BMI; weight (kg)/height (m^2^)), creatinine clearance, and nephrotoxicity. Creatinine clearance was calculated according to the Cockcroft–Gault formula (Schlobohm et al., 2021; Cockcroft and Gault, 1976). Nephrotoxicity was defined as an increase in serum creatinine concentration by more than 50% after vancomycin administration (Chung et al., 2012).

### 2.2 Vancomycin dosing regimen and monitoring of plasma trough concentration

All patients received an intravenous infusion of vancomycin 1.0 g q12 h. In this study, the steady-state blood vancomycin concentration was considered to be reached after administering the fifth dose of vancomycin (He et al., 2020). The blood vancomycin concentration was measured half an hour before administration of the sixth dose and was used as the trough concentration. The blood concentration of vancomycin was measured using chemiluminescence immunoassay analyzer (ARCHITECT PLUS i2000 SR; Abbott Laboratories, United States). The detection limit of the vancomycin monitor was 3.0–100.0 μg/mL.

### 2.3 Grouping

With reference to a previous study (He et al., 2020), enrolled patients were divided into two groups based on the blood vancomycin trough concentration, i.e., the therapeutic group had vancomycin trough concentrations within the target therapeutic range (10–20 mg/L) and the subtherapeutic group had trough concentration in the subtherapeutic range (<10 mg/L).

### 2.4 Ethics and funding

This was a retrospective study. The study was approved by the Ethics Committee of Ruijin Hospital, Shanghai Jiaotong University School of Medicine. The privacy of patient data was protected in accordance with ethical guidelines. The approval number was (2020) Clinical Ethics Review No. (221).

### 2.5 Statistical analysis

IBM SPSS 26.0 (IBM Corp., Armonk, NY, United States) was used to analyze the data. Measurement data (age, weight, height, BMI, burn area, area of third-degree burns, days between burn injury and drug administration, serum creatinine, serum albumin, APACHE II score, blood vancomycin trough concentration, creatinine clearance) are expressed as mean and standard deviation, and intergroup comparisons were performed using a *t*-test. Count data (nephrotoxicity, tracheal incision) are expressed as number and frequency, and intergroup comparisons were performed using the chi-square test. Diagnostic tests were performed using receiver operating characteristic (ROC) curves. For factors influencing the trough concentration, (age, weight, height, BMI, burn area, area of third-degree burns, days between burn injury and drug administration, serum creatinine, serum albumin, APACHE II score, creatinine clearance), Pearson correlation analysis was used in correlation analysis, then linear stepwise regression was used in regression analysis. We considered *p* < 0.05 to indicate statistical significance.

## 3 Results

### 3.1 Basic patient information

As shown in Table 1, a total of 80 patients were enrolled. Among included patients, the average age was 45.08 ± 14.59 years, the average weight was 65.52 ± 12.12 kg, the average burn area was 34.15% ± 28.57% total body surface area (%TBSA), the average area of third-degree burns was 12.51 ± 19.22%TBSA, and the average vancomycin trough concentration was 6.79 ± 4.47 μg/mL.

**TABLE 1:** Basic patient information.

Age (years)	45.08 ± 14.59
Weight (kg)	65.52 ± 12.12
Height (cm)	165.55 ± 9.30
BMI	23.79 ± 3.27
Burn area (%TBSA)	34.15 ± 28.57
Area of third-degree burns (%TBSA)	12.51 ± 19.22
Days between drug administration and burn injury	7.33 ± 10.35
Serum creatinine level before drug administration (μmol/L)	66.38 ± 24.78
Serum albumin level before drug administration (g/L)	33.39 ± 5.87
APACHE II score	8.03 ± 3.65
Creatinine clearance (mL/min)	118.37 ± 44.86
Trough concentration (µg/mL)	6.79 ± 4.47
Number of patients who underwent tracheotomy	20
Number of cases of nephrotoxicity	5

BMI, body mass index; APACHE, Acute Physiology and Chronic Health Evaluation II; TBSA, total body surface area.

### 3.2 Grouping by vancomycin trough concentration

As shown in Table 2, the vancomycin trough concentration in 14 patients (17.5%) was within the therapeutic range, with a mean trough concentration of 14.36 ± 2.82 μg/mL; the vancomycin trough concentration in 66 patients was in the subtherapeutic range (82.5%), with a mean trough concentration of 5.18 ± 2.77 μg/mL.

**TABLE 2:** Grouping by vancomycin trough concentration.

	Number of patients	Trough concentration (µg/mL)
the therapeutic group	14 (17.5%)	14.36 ± 2.82
the subtherapeutic group	66 (82.5%)	5.18 ± 2.77

### 3.3 Group comparisons and ROC analysis of vancomycin trough concentration

As shown in Table 3, patients’ serum creatinine levels were significantly higher in the therapeutic group (84.93 ± 47.26 μmol/L) than those in the subtherapeutic group (62.44 ± 14.49 μmol/L) (*t* = −3.267, *p* = 0.002). The serum albumin level was significantly lower in the therapeutic group (30.50 ± 2.28 g/L) than that in the subtherapeutic group (34.00 ± 6.22 g/L) (*t* = 2.068, *p* = 0.042); creatinine clearance was also significantly lower in the therapeutic group (88.00 ± 46.97 mL/min) than that in the subtherapeutic group (124.81 ± 42 mL/min) (*t* = 2.918, *p* = 0.005). Table 4 shows that for serum albumin level, the AUC (95% CI) was 0.67 (0.553, 0.788) and the optimal cut-off point was 34.50 g/L (*p* = 0.046); for creatinine clearance, the AUC (95% CI) was 0.72 (0.537, 0.902), and the optimal cut-off point was 76.64 mL/min (*p* = 0.01).

**TABLE 3:** Comparison of data between the two groups.

	The subtherapeutic group		The therapeutic group		t/χ^2^ value	*p*-value
Age (years)	43.79	±12.9	51.14	±20.34	−1.735	0.087
Weight (kg)	66.51	±12.21	60.86	±10.94	1.6	0.114
Height (cm)	165.88	±9.51	164.00	±8.38	0.684	0.496
BMI	24.07	±3.35	22.47	±2.57	1.679	0.097
Burn area (%)	32.83	±26.84	40.29	±36.08	−0.884	0.379
Area of third-degree burns (%)	10.85	±17.34	20.36	±25.73	−1.701	0.093
Days between drug administration and burn injury (days)	6.94	±9.64	9.14	±13.48	−0.722	0.473
Serum creatinine level before drug administration (μmol/L)	62.44	±14.49	84.93	±47.26	−3.267	0.002
Serum albumin level before drug administration (g/L)	34.00	±6.22	30.50	±2.28	2.068	0.042
APACHE II score	7.95	±3.47	8.36	±4.53	−0.373	0.71
Creatinine clearance (mL/min)	124.81	±42	88.00	±46.97	2.918	0.005
Tracheotomy						
None	51 (77.30)		9 (64.30)		1.039	0.308
Yes	15 (22.70)		5 (35.70)			
Nephrotoxicity						
None	62 (93.90)		13 (92.90)		0.023	0.879
Yes	4 (6.10)		1 (7.10)			

BMI, body mass index; APACHE, Acute Physiology and Chronic Health Evaluation II.

**TABLE 4:** ROC diagnosis of standard vancomycin trough concentration.

Indicators	AUC	Standard error	*p*-value	Optimal cut-off point	Sensitivity	Specificity	Youden index
Serum albumin level before drug administration (g/L)	0.67 (0.553, 0.788)	0.06	0.046	34.50	0.379	1	0.379
Creatinine clearance (mL/min)	0.72 (0.537, 0.902)	0.093	0.01	76.64	0.985	0.5	0.485

ROC, receiver operating characteristic; AUC, area under the ROC, curve.

### 3.4 Correlation analysis and linear stepwise regression analysis of vancomycin trough concentration

As seen in Table 5, the vancomycin trough concentration was positively correlated with age (r = 0.419, *p* < 0.001), area of third-degree burns (r = 0.295, *p* = 0.007), days between burn injury and drug administration (r = 0.291, *p* = 0.008), and serum creatinine level (r = 0.412, *p* < 0.001). The vancomycin trough concentration was negatively correlated with serum albumin level (r = −0.281, *p* = 0.01) and creatinine clearance (r = −0.512, *p* < 0.001). As seen in Table 6, age (*t* = 4.954, *p* < 0.001) and serum creatinine level (*t* = 4.256, *p* < 0.001) had significant positive effects on the trough concentrations. The linear stepwise regression equation was as follows: trough concentration = 0.14 × age + 0.071 × serum creatinine −4.196.

**TABLE 5:** Correlation analysis of vancomycin trough concentrations.

Indicators	r coefficient	*p*-value
Age (years)	0.419	<0.001
Weight (kg)	−0.207	0.061
Height (cm)	−0.131	0.238
BMI	−0.171	0.122
Burn area (%)	0.132	0.237
Area of third-degree burns (%)	0.295	0.007
Days between drug administration and burn injury (days)	0.291	0.008
Serum creatinine level before drug administration (μmol/L)	0.412	<0.001
Serum albumin level before drug administration (g/L)	−0.281	0.01
APACHE II score	0.184	0.097
Creatinine clearance (mL/min)	−0.512	<0.001

BMI, body mass index; APACHE, Acute Physiology and Chronic Health Evaluation II.

**TABLE 6:** Linear stepwise regression analysis of vancomycin trough concentrations.

Indicators	B	Standard error	Beta	t value	*p*-value
(constant)	−4.196	1.761		−2.382	0.02
Age (years)	0.14	0.028	0.456	4.954	<0.001
Serum creatinine level before drug administration (μmol/L)	0.071	0.017	0.392	4.256	<0.001

## 4 Discussion

Infection has always been a problem in the treatment of burn patients (Fitzwater et al., 2003). *S. aureus*, especially methicillin-resistant *S. aureus* is one of the main pathogens detected in patients with burn injury (Guggenheim et al., 2009; Hu et al., 2021). Vancomycin is a first-choice glycopeptide antibiotic (Levine, 2006) and is used worldwide for the treatment of infection with methicillin-resistant *S. aureus* (Liu et al., 2011). However, substandard blood drug concentrations can lead to vancomycin treatment failure and the development of drug-resistant bacteria (Ravina et al., 2011; Pea et al., 2005). Therefore, recommendations regarding the blood vancomycin trough concentration have been established (He et al., 2020). However, there is scant PK/PD research involving burn patients; therefore, further investigation is needed on changes in the PK/PD parameters when using antibiotics, including vancomycin, in this patient population (Carter et al., 2015; Pruskowski, 2021; Lee et al., 2017; Stanojcic et al., 2018). Among vancomycin-related PK/PD parameters, although the AUC24 is more suitable than trough concentration as a monitoring indicator (He et al., 2020), vancomycin AUC24 values for burn patients require multiple blood drug concentration measurements and are not easy to obtain (Rybak et al., 2009; Holmes, 2020). Because trough concentration and AUC24 are highly consistent (Ayuthaya et al., 2020), trough concentration can also be used as a monitoring indicator (He et al., 2020; Neely et al., 2018). Therefore, this study was conducted to further understand influencing factors of the blood vancomycin trough concentrations in burn patients and to provide a basis for how to better achieve effective blood vancomycin concentrations in this patient population.

In this study, the vancomycin trough concentration was within the standard range in only 14 of 80 burn patients (17.5%). ROC curve analysis conducted using the standard vancomycin trough concentration indicated that when albumin levels were greater than 34.50 g/L, the vancomycin trough concentration in burn patients was likely to not be within the standard range; also, when creatinine clearance was less than 76.64 mL/min, the vancomycin trough concentration of burn patients was more likely to be within the standard range. Among these two indicators, creatinine clearance showed a better predictive effect. Correlation analysis of influencing factors showed that the vancomycin trough concentration was positively correlated with age, area of third-degree burns, days between burn injury and drug administration, and serum creatinine level; the trough concentration was negatively correlated with serum albumin level and creatinine clearance. Further linear stepwise regression analysis was performed and a linear progressive regression equation was obtained: trough concentration = 0.14 × age + 0.071 × serum creatinine −4.196. As our study findings confirmed, a high proportion of burn patients with a vancomycin trough concentration below the standard range is common (Ayuthaya et al., 2020; Carter et al., 2015; Pruskowski, 2021; Elligsen et al., 2011). This phenomenon is owing to the unique pathophysiological changes in these patients (Pruskowski, 2021). Within 48–72 h after the initial burn injury, patients with severe burns may go into burn shock and require massive fluid resuscitation; vascular permeability and tissue edema increase; hypoproteinemia, myocardial contractility, and cardiac output decrease; and blood flow to the intestines, liver, and kidneys declines. In the middle and late stages of burn injury, patients can develop hypermetabolic responses; systemic inflammation and oxidative stress lead to increased cardiac contractility and cardiac output; and blood flow to the liver and kidneys increases, thereby increasing hepatic metabolism and renal clearance. The unique biphasic changes over time can cause the PK/PD parameters (apparent volume of distribution, protein binding, renal clearance, etc.,) of burn patients to be affected by various factors (Carter et al., 2015), including age, co-morbidities, fluid resuscitation, concurrent administration of nephrotoxic drugs, burn area, existence of inhalation injuries, and time between administration of vancomycin and burn injury. Therefore, if the PK/PD parameters of nonburn patients are used to develop antibiotic regimens for patients with burn injury, the effective drug concentration may not be achieved in the latter group of patients (Lee et al., 2017; Musick et al., 2022), resulting in treatment failure. After vancomycin enters the body, some of the drug binds to albumin and some is excreted through the kidneys. Therefore, serum albumin levels and kidney function play an important role in vancomycin metabolism. Our study also validated this point, showing that in ROC curve analysis, serum albumin and creatinine clearance are important parameters to predict whether vancomycin concentration is within the standard range. Our linear regression equation additionally showed that the trough concentration of vancomycin in burn patients can be predicted using age and serum creatinine. One study (Lin et al., 2021) reported that the apparent volume distribution of vancomycin decreased with increased age and serum creatinine level, leading to high blood concentrations. As shown in our linear regression equation, the higher the age and serum creatinine level, the higher the trough concentration of vancomycin. This formula facilitates the use of age and serum creatinine values to estimate the trough concentration in burn patients when this concentration cannot be measured in a timely manner, thereby providing a means for more effective anti-infective therapy. However, in our stepwise regression analysis, only age and serum creatinine levels were included in the regression equation; the previously discussed serum albumin was not included in the equation. The fact that serum albumin was excluded from the final equation in multiple regression analysis suggests that this variable may be colinear with other confounding factors, and its addition to the predictivity of the model is not statistically significant.

Serum creatinine levels were significantly higher in the therapeutic group than those in the subtherapeutic group; serum albumin levels were significantly lower in the therapeutic group than those in the subtherapeutic group; and creatinine clearance levels were significantly lower in the therapeutic group than those in the subtherapeutic group. Age, height, weight, BMI, burn area, area of third-degree burns, days between burn injury and drug administration, APACHE II score and incidence of nephrotoxicity were not significantly different between the two groups. Vancomycin is a hydrophilic drug that is mainly excreted via the kidneys; therefore, its clearance is closely related to creatinine clearance (Baptista et al., 2012). Because the use of conventional doses may lead to insufficient blood drug concentrations, high creatinine clearance has been considered an important cause of treatment failure with renally cleared antibiotics such as β-lactams and vancomycin (Cook and Hatton-Kolpek, 2019; Udy et al., 2012) in critically ill patients and burn patients. In a previous study (Baptista et al., 2012), a good correlation was found between vancomycin clearance and creatinine clearance; vancomycin blood concentrations were low in patients with hyperactive renal function (i.e., augmented renal clearance), and subtherapeutic concentrations of vancomycin were detected when the standard dosage was used. Studies have reported that the appropriate dose of vancomycin is dependent on creatinine clearance (Tsai et al., 2018). In our study, reaching the target blood vancomycin trough concentration was closely related to creatinine clearance. The vancomycin trough concentration is related to the patient’s actual body weight (Tsai et al., 2018); however, our research showed that weight and BMI are not related to the trough concentration. This conclusion may because our sample size (N = 80) may not have been sufficiently large; further validation is needed in future research.

The results of a previous study (Lin et al., 2021) indicated that serum creatinine is negatively correlated with vancomycin clearance. In our study, the serum creatinine level before drug administration was significantly higher in the therapeutic group than that in the subtherapeutic group. There was no significant difference in renal toxicity between the two groups after treatment. Therefore, we believe that a higher initial serum creatinine concentration facilitates reaching the target vancomycin trough concentration.

Albumin plays an important role in drug PK. After drugs enter the bloodstream, a portion binds to albumin and another portion remains as free drug, exerting pharmacological effects and being metabolized by the body, such as in the liver and kidney. Thus, hypoproteinemia can lead to an increase in free drug and increased drug clearance. Studies have shown that hypoproteinemia may lead to an increase in the clearance of some antibiotics such as ceftriaxone in critically ill patients, resulting in decreasing drug concentrations and failure to achieve the PD goals associated with efficacy (Ulldemolins et al., 2011). Vancomycin is hydrophilic, and the protein binding rate is approximately 50%–55% (Porter, 2023). There are few studies regarding the effect of albumin levels on blood vancomycin concentrations. Furthermore, to our knowledge, no studies have examined the effect of albumin level on the blood concentration of vancomycin in burn patients. In a previous sepsis study (Kovacevic et al., 2019), the vancomycin trough concentration in patients with severe sepsis who had severe hypoproteinemia (<25 mg/L) was significantly higher than that in their counterparts with non-severe hypoproteinemia (>25 mg/L). One study showed that the half-life of vancomycin in older patients with hypoproteinemia was significantly longer than that in patients without hypoalbuminemia (Tomohiro et al., 2013), possibly because hypoproteinemia can affect free vancomycin, resulting in a prolonged half-life. Our study of burn patients also seemed to confirm the findings of previous studies (Kovacevic et al., 2019; Tomohiro et al., 2013), that lower albumin levels may lead to an increase in the vancomycin trough concentration. This phenomenon is opposite to the effect of hypoalbuminemia on the concentration of certain antibiotics (such as ceftriaxone); that is, the lower the albumin level, the more free drug and the higher the clearance rate of ceftriaxone (Ulldemolins et al., 2011). More importantly, however, we found that the therapeutic group of patients not only had a lower albumin level, they also had a lower creatinine clearance. Combined with these two indicators, we believe that the increase in vancomycin concentration among burn patients with hypoproteinemia has an important relationship with the decrease in creatinine clearance. Low albumin levels can lead to increased levels of free vancomycin in plasma, which may lead to increased renal excretion and decreased drug concentrations with normal renal function. However, when patients with low albumin have low creatinine clearance, increasing free drug does not increase drug excretion; instead, this increases the drug concentration owing to decreased renal clearance. This also suggests that greater attention is needed regarding changes in renal function when it is suspected that hypoproteinemia affects the blood concentration. For patients with normal or hyperactive renal function, free drug excreted via the kidneys is increased, and hypoproteinemia may lead to a decrease in the plasma drug concentration. In patients with impaired renal function, less free drug is excreted through the kidneys, and hypoproteinemia does not result in a decrease or even an increase in the drug concentration. In our linear stepwise regression equation, albumin was eliminated and renal function retained, indicating that albumin may be collinear with other factors.

In a previous report (Elder et al., 2017), the closer the timing of drug administration to the day of the initial burn injury and the larger the burn area, the higher the vancomycin clearance and the higher the required daily dose of vancomycin. However, in our study, we did not observe this phenomenon. It is generally agreed that burn area is one parameter used to evaluate the severity of a patient’s condition; the larger the burn area, the more severe the patient’s condition, and the lower the trough concentration of vancomycin. After the initial burn injury occurs, there may be capillary leakage and augmented renal clearance, which will lead to a reduction in the vancomycin concentration. This condition may gradually improve with an increased number of days after the burn injury occurs (Pruskowski, 2021). Therefore, the longer the time since the initial injury, the better the patient’s condition, and the higher the trough concentration of vancomycin. However, the reason why our research is inconsistent with the findings of other studies may be that neither the burn area nor the number of days after the occurrence of burn injury directly affect the drug concentration. These certainly affect the drug concentration by affecting factors such as albumin or kidney function. However, patients differ, so even among patients with the same burn area or the same number of days after a burn injury, physiological indicators such as creatinine clearance and plasma albumin may differ. Therefore, we did not find an effect on drug concentrations of the burn area or time between burn injury and drug administration. Compared with the burn area or number of days after burn injury, plasma albumin or renal function may be more accurate as factors influencing the vancomycin trough concentration.

### 4.1 Limitations of this study

Several study limitations should be mentioned. First, this was a retrospective study and the data were obtained by reviewing patients’ electronic medical records. Second, we only investigated relevant factors influencing the trough concentration and did not explore factors influencing the AUC24. Third, this was a single-center study, and the results may not directly apply to patients in other burn centers. Fourth, the study results only pertain to adult patients, not to pediatric patients. Fifth, the sample size of 80 patients was not large; we will include additional patients in future studies to validate the equation derived in this study. Sixth, we only included patients with a dosing regimen of 1 g q12 h; thus, we limited the predictive variables to age and creatinine clearance without including the dosing regimen. Therefore, this study could not address the question of how to adjust dosages when the predicted concentrations are low.

### 4.2 Highlights of this study

Despite the above limitations, we found that compared with albumin, renal function was more important in influencing the vancomycin drug concentration among burn patients. Contrary to the results of previous studies, factors such as the number of days between burn injury and drug administration as well as burn area had no effect on the vancomycin concentration in our study. Additionally, a linear stepwise regression equation was obtained for the trough concentration; this equation can be used to estimate the trough concentration using the patient’s age and serum creatinine level.

### 4.3 Conclusion

With a dosing regimen of 1.0 g q12 h, a low proportion of adult burn patients in our study had blood vancomycin trough concentrations within the standard range. In the therapeutic group, patients’ albumin levels were lower and their serum creatinine levels were higher. Serum creatinine clearance and albumin levels are important indicators for predicting whether a patient’s blood vancomycin trough concentration is within the standard range. According to the linear stepwise regression equation, when the trough concentration cannot be measured in a timely manner for burn patients with infection, age and serum creatinine can be used to estimate the vancomycin trough concentration.

## Data Availability

The raw data supporting the conclusions of this article will be made available by the authors, without undue reservation.

## References

[B1] Aljutayli A. Amélie M. Nekka F. (2020). An update on population pharmacokinetic analyses of vancomycin, Part I: in adults. Clin. Pharmacokinet. 59 (6), 671–698. 10.1007/s40262-020-00866-2 32020531

[B2] Álvarez O. Plaza-Plaza J. C. Ramirez M. Peralta A. Amador C. A. Amador R. (2017). Pharmacokinetic assessment of vancomycin loading dose in critically ill patients. Antimicrob. Agents Chemother. 61, 00280–e317. 10.1128/AAC.00280-17 PMC5527600 28607023

[B3] Ayuthaya S. I. N. Katip W. Oberdorfer P. Lucksiri A. (2020). Correlation of the vancomycin 24-h area under the concentration-time curve (AUC24) and trough serum concentration in children with severe infection: a clinical pharmacokinetic study. Int. J. Infect. Dis. 92, 151–159. 10.1016/j.ijid.2019.12.036 31935538

[B4] Baptista J. P. Sousa E. Martins P. J. Pimentel J. M. (2012). Augmented renal clearance in septic patients and implications for vancomycin optimisation. Int. J. Antimicrob. Agents 39 (5), 420–423. 10.1016/j.ijantimicag.2011.12.011 22386742

[B5] Carter B. L. Damer K. M. Walroth T. A. Buening N. R. Foster D. R. Sood R. (2015). A systematic review of vancomycin dosing and monitoring in burn patients. J. Burn Care Res. 36, 641–650. 10.1097/BCR.0000000000000191 25423436

[B6] Chu Y. Luo Y. Jiang M. Zhou B. (2019). Application of vancomycin in patients with augmented renal clearance. Eur. J. Hosp. Pharm. Sci. Pract. 27 (5), 276–279. 10.1136/ejhpharm-2018-001781 PMC7447238 32839259

[B7] Chu Y. Luo Y. Qu L. Zhao C. Jiang M. (2016). Application of vancomycin in patients with varying renal function, especially those with augmented renal clearance. Pharm. Biol. 54, 2802–2806. 10.1080/13880209.2016.1183684 27251880

[B8] Chung K. K. Stewart I. J. Gisler C. Simmons J. W. Aden J. K. Tilley M. A. (2012). The Acute kidney injury network (AKIN) criteria applied in burns. J. Burn Care Res. 33 (4), 483–490. 10.1097/BCR.0b013e31825aea8d 22688091

[B9] Cockcroft D. W. Gault H. (1976). Prediction of creatinine clearance from serum creatinine. Nephron 16 (1), 31–41. 10.1159/000180580 1244564

[B10] Cook A. M. Hatton-Kolpek J. (2019). Augmented renal clearance. Pharmacotherapy 39 (3), 346–354. 10.1002/phar.2231 30723936

[B12] Dou Y. Huan J. Guo F. Zhou Z. Shi Y. (2017). *Pseudomonas aeruginosa* prevalence, antibiotic resistance and antimicrobial use in Chinese burn wards from 2007 to 2014. J. Int. Med. Res. 45 (3), 1124–1137. 10.1177/0300060517703573 28443385 PMC5536433

[B13] Elder K. Hill D. M. Hickerson W. L. (2017). Characterization of variables for potential impact on vancomycin pharmacokinetics in thermal or inhalation injury. Burns 44, 658–664. 10.1016/j.burns.2017.10.004 29097070

[B14] Elligsen M. Walker S. A. N. Walker S. E. Simor A. (2011). Optimizing initial vancomycin dosing in burn patients. Burns 37 (3), 406–414. 10.1016/j.burns.2010.06.005 21095062

[B15] Elyasi S. Khalili H. Dashti-Khavidaki S. Mohammadpour A. (2012). Vancomycin-induced nephrotoxicity: mechanism, incidence, risk factors and special populations. A literature review. Eur. J. Clin. Pharmacol. 68 (9), 1243–1255. 10.1007/s00228-012-1259-9 22411630

[B16] Fitzwater J. Purdue G. F. Hunt J. L. O'Keefe G. E. (2003). The risk factors and time course of sepsis and organ dysfunction after burn trauma. J. Trauma Acute Care Surg. 54, 959–966. 10.1097/01.TA.0000029382.26295.AB 12777910

[B17] Greenhalgh D. G. Saffle J. R. Holmes J. H. Gamelli R. L. Palmieri T. L. Horton J. W. (2007). American burn association consensus conference to define sepsis and infection in burns. J. Burn Care Res. 28 (6), 776–790. 10.1097/BCR.0b013e3181599bc9 17925660

[B18] Guggenheim M. Zbinden R. Handschin A. E. Gohritz A. Altintas M. A. Giovanoli P. (2009). Changes in bacterial isolates from burn wounds and their antibiograms: a 20-year study (1986–2005). Burns 35 (4), 553–560. 10.1016/j.burns.2008.09.004 19167827

[B19] He N. Su S. Ye Z. Du G. He B. Li D. (2020). Evidence-based guideline for therapeutic drug monitoring of vancomycin: 2020 update by the division of therapeutic drug monitoring, Chinese pharmacological society. Clin. Infect. Dis. 71 (Suppl. 4), S363–S371. 10.1093/cid/ciaa1536 33367582

[B21] Hill D. M. Velamuri S. R. Lanfranco J. Romero Legro I. Sinclair S. E. Hickerson W. L. (2018). Optimization of an empiric vancomycin dosing algorithm for improved target concentration attainment in patients with thermal injury. Burns 45, 423–432. 10.1016/j.burns.2018.09.025 30340863

[B22] Holmes N. E. (2020). Using AUC/MIC to guide vancomycin dosing: ready for prime time? Clin. Microbiol. Infect. 26 (4), 406–408. 10.1016/j.cmi.2019.12.023 31927116

[B23] Hu Y. Li D. Xu L. Hu Y. Sang Y. Zhang G. (2021). Epidemiology and outcomes of bloodstream infections in severe burn patients: a six-year retrospective study. Antimicrob. Resist. Infect. Control 10 (1), 98. 10.1186/s13756-021-00969-w 34193300 PMC8243830

[B24] Kovacevic T. Miljkovic B. Mikov M. Stojisavljevic Satara S. Dragic S. Momcicevic D. (2019). The effect of hypoalbuminemia on the therapeutic concentration and dosage of vancomycin in critically ill septic patients in low-resource countries. Dose-Response 17 (2), 1559325819850419. 10.1177/1559325819850419 31205457 PMC6537498

[B25] Lee C. Walker S. A. N. Walker S. E. Seto W. Simor A. Jeschke M. (2017). A prospective study evaluating tobramycin pharmacokinetics and optimal once daily dosing in burn patients. Burns 43 (8), 1766–1774. 10.1016/j.burns.2017.05.009 28647460

[B26] Levine D. P. (2006). Vancomycin: a history. Clin. Infect. Dis. 42 (1), S5–S12. 10.1086/491709 16323120

[B27] Lin Z. Chen D. Y. Zhu Y. W. Jiang Z. L. Cui K. Zhang S. (2021). Population pharmacokinetic modeling and clinical application of vancomycin in Chinese patients hospitalized in intensive care units. Sci. Rep. 11 (1), 2670. 10.1038/s41598-021-82312-2 33514803 PMC7846798

[B28] Liu C. Bayer A. Cosgrove S. E. Daum R. S. Fridkin S. K. Gorwitz R. J. (2011). Clinical practice guidelines by the infectious diseases society of America for the treatment of methicillin-resistant *Staphylococcus aureus* infections in adults and children: executive summary. Clin. Infect. Dis. 52, 285–292. 10.1093/cid/cir034 21217178

[B29] Musick K. L. Jones S. L. Norris A. M. Hochstetler L. J. Williams F. N. McKinzie B. P. (2022). Evaluation of voriconazole and posaconazole dosing in patients with thermal burn injuries. J. Burn Care Res. 43 (4), 802–807. 10.1093/jbcr/irab200 34672325 PMC9391605

[B30] Neely M. N. Kato L. Youn G. Kraler L. Bayard D. van Guilder M. (2018). Prospective trial on the use of trough concentration versus area under the curve to determine therapeutic vancomycin dosing. Antimicrob. Agents Chemother. 62 (2), 02042–e2117. 10.1128/AAC.02042-17 PMC5786789 29203493

[B31] Nham E. Huh K. Sohn Y. M. Park H. J. Kim H. Woo S. Y. (2022). Pharmacokinetic/pharmacodynamic parameters of vancomycin for predicting clinical outcome of enterococcal bacteremia. BMC Infect. Dis. 22 (1), 686. 10.1186/s12879-022-07668-w 35948963 PMC9364583

[B32] Ortwine J. K. Pogue J. M. Janie F. (1900). Pharmacokinetics and pharmacodynamics of antibacterial and antifungal agents in adult patients with thermal injury: a review of current literature. J. Burn Care Res. 36 (2), 72–84. 10.1097/BCR.0000000000000147 25167375

[B34] Pea F. Viale P. Furlanut M. (2005). Antimicrobial therapy in critically ill patients: a review of pathophysiological conditions responsible for altered disposition and pharmacokinetic variability. Clin. Pharmacokinet. 44 (10), 1009–1034. 10.2165/00003088-200544100-00002 16176116

[B35] Porter S. A. (2023). Supratherapeutic vancomycin concentrations associated with hypothermia in a burn patient. J. Burn Care Res. 39 (6), 1058–1063. 10.1093/jbcr/irx038 29931313

[B36] Pruskowski K. A. (2021). Pharmacokinetics and pharmacodynamics of antimicrobial agents in burn patients. Surg. Infect. (Larchmt) 22 (1), 77–82. 10.1089/sur.2020.375 33164665

[B37] Ravina K. Davis S. L. Levine D. P. Rybak M. J. (2011). Impact of vancomycin exposure on outcomes in patients with methicillin-resistant *Staphylococcus aureus* bacteremia: support for consensus guidelines suggested targets. Clin. Infect. Dis. 52 (8), 975–981. 10.1093/cid/cir124 21460309

[B38] Rybak M. J. Lomaestro B. M. Rotscahfer J. C. Moellering R. C. Jr. Craig W. A. Billeter M. (2009). Vancomycin therapeutic guidelines: a summary of consensus recommendations from the infectious diseases society of America, the American society of health-system pharmacists, and the society of infectious diseases pharmacists. Clin. Infect. Dis. 49 (3), 325–327. 10.1086/600877 19569969

[B39] Schlobohm C. Zhu E. Duby J. (2021). Continuous infusion versus intermittent infusion vancomycin in a burn center intensive care unit. Burns 47 (7), 1495–1501. 10.1016/j.burns.2021.08.016 34538672

[B40] Stanojcic M. Abdullahi A. Rehou S. Parousis A. Jeschke M. G. (2018). Pathophysiological response to burn injury in adults. Ann. Surg. 267 (3), 576–584. 10.1097/SLA.0000000000002097 29408836 PMC8966302

[B41] Tomohiro M. Fumihiro M. Kazuhiro F. Ito K. Shibasaki M. Nagamatsu T. (2013). The influence of severe hypoalbuminemia on the half-life of vancomycin in elderly patients with methicillin-resistant *Staphylococcus aureus* hospital-acquired pneumonia. Clin. Interventions Aging 8, 1323–1328. 10.2147/CIA.S52259 PMC3793010 24109180

[B42] Tsai D. Stewart P. C. Hewagama S. Krishnaswamy S. Wallis S. C. Lipman J. (2018). Optimised dosing of vancomycin in critically ill Indigenous Australian patients with severe sepsis. Anaesth. Intensive Care 46 (4), 374–380. 10.1177/0310057X1804600405 29966110

[B43] Udy A. A. Roberts J. A. Lipman J. (2011). Implications of augmented renal clearance in critically ill patients. Nat. Rev. Nephrol. 7, 539–543. 10.1038/nrneph.2011.92 21769107

[B44] Udy A. A. Varghese J. M. Altukroni M. Briscoe S. McWhinney B. C. Ungerer J. P. (2012). Subtherapeutic initial β-lactam concentrations in select critically ill patients: association between augmented renal clearance and low trough drug concentrations. Chest 142 (1), 30–39. 10.1378/chest.11-1671 22194591

[B45] Ulldemolins M. Roberts D. J. A. Rello J. Paterson D. L. Lipman J. (2011). The effects of hypoalbuminaemia on optimizing antibacterial dosing in critically ill patients. Clin. Pharmacokinet. 50 (2), 99–110. 10.2165/11539220-000000000-00000 21142293

[B46] Wu G. Xiao Y. Wang C. Hong X. Sun Y. Ma B. (2017). Risk factors for Acute kidney injury in patients with burn InjuryA meta-analysis and systematic review. J. Burn Care Res. 38 (5), 271–282. 10.1097/BCR.0000000000000438 27617407

[B47] Zhi-Kang Y. Hui-Lin T. Suo-Di Z. (2013). Benefits of therapeutic drug monitoring of vancomycin: a systematic review and meta-analysis. Plos One 8 (10), e77169. 10.1371/journal.pone.0077169 24204764 PMC3799644

